# Building the Evidence Base for Remote Data Collection in Low- and Middle-Income Countries: Comparing Reliability and Accuracy Across Survey Modalities

**DOI:** 10.2196/jmir.7331

**Published:** 2017-05-05

**Authors:** Abigail R Greenleaf, Dustin G Gibson, Christelle Khattar, Alain B Labrique, George W Pariyo

**Affiliations:** ^1^ Johns Hopkins Bloomberg School of Public Health Department of Population, Family and Reproductive Health Baltimore, MD United States; ^2^ Johns Hopkins Bloomberg School of Public Health Department of International Health Baltimore, MD United States

**Keywords:** mHealth, developing countries, Africa South of the Sahara, cell phones, health surveys, reproducibility of results, surveys and questionnaires, text messaging, interviews as topic, humans, research design, data collection methods

## Abstract

**Background:**

Given the growing interest in mobile data collection due to the proliferation of mobile phone ownership and network coverage in low- and middle-income countries (LMICs), we synthesized the evidence comparing estimates of health outcomes from multiple modes of data collection. In particular, we reviewed studies that compared a mode of remote data collection with at least one other mode of data collection to identify mode effects and areas for further research.

**Objective:**

The study systematically reviewed and summarized the findings from articles and reports that compare a mode of remote data collection to at least one other mode. The aim of this synthesis was to assess the reliability and accuracy of results.

**Methods:**

Seven online databases were systematically searched for primary and grey literature pertaining to remote data collection in LMICs. Remote data collection included interactive voice response (IVR), computer-assisted telephone interviews (CATI), short message service (SMS), self-administered questionnaires (SAQ), and Web surveys. Two authors of this study reviewed the abstracts to identify articles which met the primary inclusion criteria. These criteria required that the survey collected the data from the respondent via mobile phone or landline. Articles that met the primary screening criteria were read in full and were screened using secondary inclusion criteria. The four secondary inclusion criteria were that two or more modes of data collection were compared, at least one mode of data collection in the study was a mobile phone survey, the study had to be conducted in a LMIC, and finally, the study should include a health component.

**Results:**

Of the 11,568 articles screened, 10 articles were included in this study. Seven distinct modes of remote data collection were identified: CATI, SMS (singular sitting and modular design), IVR, SAQ, and Web surveys (mobile phone and personal computer). CATI was the most frequent remote mode (n=5 articles). Of the three in-person modes (face-to-face [FTF], in-person SAQ, and in-person IVR), FTF was the most common (n=11) mode. The 10 articles made 25 mode comparisons, of which 12 comparisons were from a single article. Six of the 10 articles included sensitive questions.

**Conclusions:**

This literature review summarizes the existing research about remote data collection in LMICs. Due to both heterogeneity of outcomes and the limited number of comparisons, this literature review is best positioned to present the current evidence and knowledge gaps rather than attempt to draw conclusions. In order to advance the field of remote data collection, studies that employ standardized sampling methodologies and study designs are necessary to evaluate the potential for differences by survey modality.

## Introduction

In low- and middle-income countries (LMICs), where vital registration, surveillance, and health record systems are underdeveloped [1], improved modes of data collection are needed [2]. Public health practitioners could benefit from more timely estimates and indicators to better plan programs, design interventions, and assess progress. The financial and human resource burden of a large survey as well as the need for more frequent data collection, particularly as mandated by the Sustainable Development Goals [3], all justify an improved system to monitor health indicators in LMICs.

The rapid increase of mobile phone ownership in LMICs offers a platform for low-cost, frequent data collection. Urbanization, increased mobile phone network coverage, and the low cost of purchasing a mobile phone have contributed to increased mobile phone ownership in LMICs [4]. According to the International Telecommunications Union, in 2015 the number of mobile subscriptions worldwide was 98.66 per 100 people [5]. Increased mobile phone ownership presents the opportunity to survey respondents remotely, whether via short message service (SMS), computer-assisted telephone interview (CATI), interactive voice response (IVR), or Web surveys. The advantages and disadvantages of these interview modalities are discussed by Gibson et al [6]. As remote data collection becomes more common in LMICs, the reliability and accuracy of data collected should be compared with established methods, including the reference-standard household survey.

There is a well-established body of literature on mobile phones as survey instruments in high-income countries [7-11], but there is a dearth of rigorous research that compares the quality, reliability, and accuracy of remote data collection modes in LMICs. Data collection mode can influence social desirability bias, can impact response rates, or can change the cognitive process for answer retrieval [9]. Although misreporting of sensitive behaviors has long been of interest to survey methodologists, a superior interviewing tool is yet to be identified for use in LMICs [12]. Cognitive models illustrate how mode of data collection affects information retrieval, judgments about the appropriate responses, and answer choices [11]. Notably, response rates are traditionally lower for remote data collection compared with face-to-face (FTF) data collection [10].

The purpose of this literature review was to identify and synthesize the available literature from LMICs that compare a mode of remote health data collection with at least one other data collection mode. We also discuss reliability and construct validity across measures. By synthesizing the research that compares a mode of remote data collection to another mode, we identify the strengths and limitations of remote modes in LMICs as well as areas for future research.

## Methods

This literature review utilized the search terms and primary inclusion and exclusion criteria from a previously conducted literature review in March and April 2015 [6]. We adapted search terms for mobile phone, IVR, text message, survey, questionnaire, and data collection to each database’s classification system to query seven databases of peer-reviewed and grey literature. The abstracts and titles were screened against a set of primary inclusion and exclusion criteria [6]. The primary inclusion criteria required that the research was collected from the respondent by SMS, IVR, CATI, or via mobile phone.

Once all articles that met the primary inclusion and exclusion criteria were identified, two of the authors (AG and CK) independently reviewed the articles using the secondary inclusion and exclusion criteria, as listed in Textbox 1.

Summary briefs, inaccessible full texts, and a number of manuscripts that used a remote form of data collection but did not compare the method against a standard were excluded. The references of articles included in this review were searched to find relevant publications that were not identified by the literature search. Surveys included in this review were not required to be nationally representative.

**Secondary inclusion and exclusion criteria.**

Secondary inclusion criteria

- Study conducted in LMIC as defined by the World Bank
- Two or more modes of data collection are compared in the study
- At least one of the data collection modes is remote
- The survey includes questions about health

Secondary exclusion criteria

- Studies without a human component to the research
- Studies that collect only adherence information or that strictly examine the use of reminders for health-seeking behaviors and outcomes 
- Studies that compare modes of facility-based surveillance data collection

**Table 1:** Categories of data collection included in this literature review.

Survey administration	Remote	In-person
Interviewer administered	CATI^a^	FTF^b^
Self-administered	IVR^c^	IVR^d^
	SMS^e,f^	SAQ^g^
	Postal SAQ	
	Web MP^h^	
	Web PC^i^	

^a^CATI: computer-assisted telephone interview.

^b^FTF: face-to-face.

^c^IVR: interactive voice response.

^d^IVR is traditionally administered remotely but can be administered in-person by the interviewer by handing a phone to the respondent that plays an IVR survey.

^e^SMS: short message service.

^f^Two forms of SMS surveys were included in this study: single sitting (survey completed at one time) and modular (an SMS sent each day until survey is completed).

^g^SAQ: self-administered questionnaire.

^i^Web PC: Web on personal computer.

^h^Web MP: Web on mobile phone.

A standardized data collection tool containing inclusion and exclusion criteria was completed by two reviewers for each screened article. Variables in the extraction form included study design, study location, data collection mode, response rates, sensitive questions, study limitations, cost, and findings. Once compiled, the two reviewers discussed any differences in their respective reviews to make final inclusion or exclusion decisions. The two reviewers relied on a third person to clarify any inclusion disagreements.

Included articles were grouped by location of respondent in relation to interviewer (remote or in-person) and by the person administering the questionnaire (self-administered or interviewer administered; see Table 1). If the participant was in a different geographic location from the interviewer while administering the survey (no FTF interaction), data collection was classified as remote. In-person data collection was defined as FTF interviewer-respondent interaction. Self-administered was defined as surveys where respondents answer without questions from the interviewer. Interviewer administered was defined as the interviewer speaking with the respondent to elicit responses. FTF surveys were defined as an in-person, interviewer-administered survey. CATI was the only form of remote interviewer-administered survey included in this literature review. IVR, SMS, Web surveys, and a self-administered questionnaire (SAQ) sent back via post were defined as remote, self-administered surveys. There were two examples of an in-person self-administered survey: SAQ and IVR administered on-site.

We reported inter-method reliability for articles that compared the same respondents across two or more modes. For articles that did not compare the same population across the two modes, we considered external construct validity, the degree to which a measure satisfies theoretical predictions about a measurement. Included articles were reviewed and marked for sensitive questions, defined as asking about a subject that is private or taboo [13]. In assessing the results, we considered not only statistical significance, but effect size, direction of difference, and potential confounding factors [11].

## Results

### Overview

**Figure 1:**
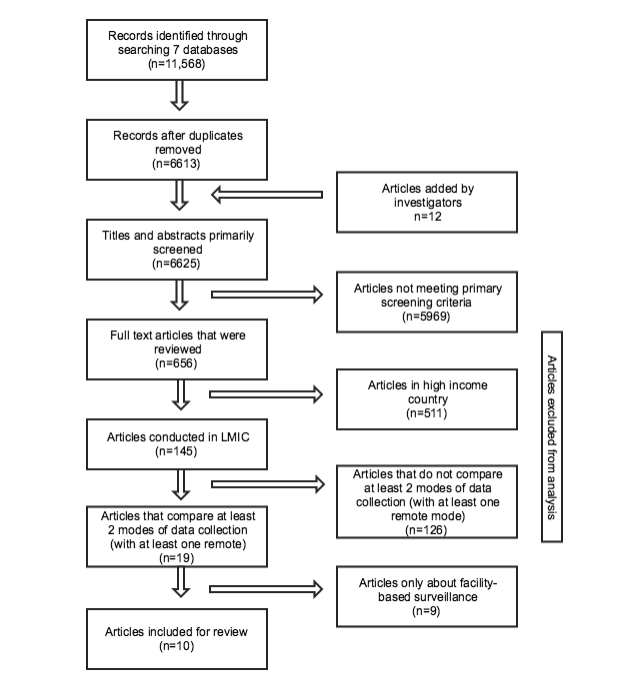
Flowchart of articles identified and included in review.

The parent systematic literature search identified 11,568 records, which after removing the duplicates and adding 6 articles identified by the authors, was reduced to 6625 records (see Figure 1). The primary inclusion and exclusion criteria further decreased the number of articles to 145. After removing 126 articles that did not include a comparison mode and 9 surveillance articles, we conducted full-text abstraction on 10 articles that compared two or more modes of data collection in a LMIC, with at least one form being remote data collection (see Table 2). All but one of the articles were published between 2011 and 2015. The 10 articles collected data in 7 countries, across 4 regions (Asia, Latin America, Europe, and the Middle East); notably none took place in Sub-Saharan Africa (SSA). One article reported data from 2 countries, Honduras and Peru [14].

Ten distinct modes of data collection were used in the 10 studies (see Table 3). The three in-person modes were FTF [14-21], SAQ, and in-person IVR [18]. The 7 types of remote data collection included two types of phone calls, IVR (remote) [14,22] and CATI [14,16,17,20,23,24]; and three modes that required respondents to type their responses into a mobile phone or computer, including SMS (all but one were singular-design) [14,15,19,21,24] and two types of Web surveys [25,26], (one administered on a personal computer (PC) and the other taken via a mobile browser on a smartphone). The most frequent mode of data collection was FTF (5/10 studies) and second most frequent was SMS (4/10 studies). The 10 articles made 25 comparisons, of which 12 were from a World Bank study in Latin America [14]. The most common comparison was FTF and CATI [14,16,17,20] (compared 5 times in 4 articles) and the second most common comparison was FTF to SMS [14,15,19] (compared 4 times in 3 articles).

The majority (8/10) of the articles collected cross-sectional data and did not provide respondents with a mobile phone; only the studies in Nepal and those in Honduras and Peru provided respondents with mobile phones [14,24]. Respondents were sampled in a variety of ways. Four of the identified articles were population-based studies, all of which enrolled participants FTF [14,16,17,20]. Five studies compared the same population across methods of data collection [14,15,19,20,25]. Finally, 6 of the 10 articles included sensitive questions [14,18,20,24,25,26].

**Table 2:** Types of data collection which are compared and the number of included studies.

Method #1	Method #2						
	CATI^a^	IVR^b^ remote	SMS^c^: singular	SMS: modular	Web MP^d^	IVR in-person	SAQ^e^ remote
CATI			1	1			
FTF^f^	5	2	4				
SAQ in-person						1	1
IVR remote	2						
Web PC^g^					2		
SAQ remote						1	
SMS: singular	2	2		1			

^a^CATI: computer-assisted telephone interview.

^f^FTF: face-to-face.

^b^IVR: interactive voice response.

^e^SAQ: self-administered questionnaire.

^c^SMS: short message service.

^g^Web PC: Web on personal computer.

^d^Web MP: Web on mobile phone.

**Table 3:** Comparison of in-person interviewer administered compared with remote interviewer administered.

Author (year)	Country (sample type)^a^	Data collection #1 (sample size)	Data collection #2 (sample size)
Ballivian [14] (2013)	Honduras (dependent)	FTF^b^ (1500)	CATI^c^ (600)
Ballivian [14] (2013)	Peru (dependent)	FTF (1500)	CATI (384)
Ferreira [16] (2011)	Brazil (independent)	FTF (4048)	CATI (440)
Francisco [17] (2011)	Brazil (independent)	FTF (2636)	CATI (2015)
Mahfoud [20] (2014)	Lebanon (dependent)	FTF (2836)	CATI (771)

^a^When participants were the same across modes, we classified the sample as dependent. Different participants across modes was labeled as an independent sample.

^b^FTF: face-to-face.

^c^CATI: computer-assisted telephone interview.

### Comparison of Modes of Data Collection

#### In-Person Interviewer Administered Compared With Remote Interviewer Administered

We identified 5 comparisons of FTF interviews with CATI surveys in 4 articles [14,16,17,20]. One article included comparisons of FTF and CATI in both Peru and Honduras [14]. Of these 5 comparisons, 2 compared responses in independent samples [16,17] and 3 used the same population across the two modalities [14,20]. All comparisons generally showed concordance of results between modes.

An independent probability sample of respondents in Brazil who were over 18, had a landline phone, and were interviewed via CATI, produced estimates similar to an independent sample of respondents who also have a landline and who answered a household survey (FTF) [16]. The respondents contacted via CATI had statistically significant different estimates for 5 (number of household residents, mean age, schooling, smoking, health insurance) of the 18 measures compared with the FTF respondents with a landline. CATI respondents when compared with all FTF respondents (regardless of landline ownership) differed on 8 of the 18 variables, but after applying poststratification weights, only three estimates were biased.

The second study in Brazil compared FTF respondents with CATI landline respondents, sampled in the same manner as the aforementioned article. Two of the four estimates (diabetes, asthma, bronchitis, or emphysema) were the same between the two samples. The other two estimates (hypertension and osteoporosis) had a higher reported prevalence among the CATI respondents [17]. Nonetheless, authors from both the studies concluded that the telephone survey was a rapid alternative to FTF surveys to provide global prevalence estimates.

After a FTF survey in Lebanon, half of the respondents were called on their mobile phones and asked an abridged version of the FTF questionnaire [20]. Overall, there was high concordance (kappa) between the CATI and FTF surveys. Kappa was above .8 for measures including age, health insurance, diabetes, current cigarette-smoking (highest agreement and kappa: agreement=95.6%, *κ*=.91), and ever cigarette-smoking (second highest agreement=93.5%, *κ*=.87). Kappa was between .6 and .8 for questions about current water-pipe smoking and past-year alcohol consumption. Reports of past-year alcohol consumption was slightly higher via CATI compared with FTF [20]. The authors concluded that estimates from the modes are reasonably comparable when data were stratified by age, gender, and education and that the difference in past-year alcohol consumption may be caused by social desirability bias [20].

The World Bank’s study in Honduras and Peru aimed to validate a survey across four modalities: FTF, IVR, CATI, and SMS [14]. The study enrolled households who answered a 7-question FTF survey on household assets and poverty into a panel survey. The survey included questions about water and sanitation. Participants in Peru were sampled using the National Statistics office sampling frame, and households below the poverty line were oversampled. Honduras also used probabilistic sampling but used the Gallup World Poll Sampling Frame and did not oversample households below the poverty line. Regardless of mode, attrition was highest among less educated, less affluent, older, rural participants [14]. In both the countries, CATI estimates were very similar to the estimates collected FTF. Compared with the FTF survey, discordant responses from the panel in Honduras ranged from −2.1% to 0% for CATI (unreported for Peru). Furthermore, CATI had the lowest discordance with the FTF survey compared with SMS and IVR [14].

#### In-Person Interviewer Administered Compared With Remote Self-Administered

Three articles made 6 comparisons of in-person interviewer administered (all FTF) and either IVR or SMS (see Table 4). Two articles from China, both about infant feeding practices, used the test-retest method to compare FTF and SMS surveys [15,19]. One of the articles began with a FTF interview then followed up with a SMS survey [15], and the other article interviewed participants in the opposite order [19]. Both articles administered the second survey after a short time period (less than 24 h and less than 3 days). The study that sent 10 text messages to participants then followed up with FTF surveys had moderate to good agreement and 62.4% of questions had the same answers for both surveys [19]. All but one kappa and inter-class correlation were between .56 and .76; (the outlier kappa=.23 was for a question about the usefulness of a feeding calendar). The last question, which was a multiple-choice categorical question, about the source of feeding knowledge, had the highest agreement (85% of the 33 responses were the same across methods) and a kappa value of .76.

Data agreement in the other Chinese feeding study was inconsistent [15]. The highest agreement was a kappa of .86 for the first question on the survey which was about breastfeeding the day before. The other 4 questions had moderate to poor agreement. Data agreement was worst for dietary recall, with a kappa ranging from .02 to .36 for the 7 food categories. The authors proposed that certain terms were difficult for mothers to understand (eg, iron-fortified food, solid or semi-solid food) and that during the FTF survey, the interviewers could explain these concepts, a feat that SMS cannot achieve due to the limited characters in a text and constraints on the number of texts a respondent is willing to receive. Du et al also explored the length of time between the two surveys (3 hours compared to 8 hours) but did not find a statistically significant difference in reported measure by the length of time between surveys.

The World Bank study in Honduras and Peru compared FTF with two modes of remote self-administered: IVR and SMS. Compared with the FTF survey, discordant responses from the panel in Honduras ranged from −14.6% to 12.7% for IVR, and from −15.6% to 15.3% for SMS. Discordant responses were similar for IVR and SMS on a per question basis. The IVR and SMS responses were statistically significantly different from the FTF estimates. To assess the reliability, the same respondents were asked a question second time, within 10 weeks of the first administration of the questions. The total reliability coefficient in Honduras for SMS and FTF were quite similar (.74 and .77, respectively). IVR had the highest reliability coefficient (.86); but because the IVR results were most discordant with the other modes, the authors concluded that IVR was not a suitable mode for this survey. However, authors were satisfied with the reliability of SMS surveys in their study’s context.

#### In-Person Self-Administered Compared With Remote Self-Administered

To assess human immunodeficiency virus (HIV)–related risk behaviors among Hong Kong migrant men who were aged 18-60 years, authors systematically sampled 2416 migrants at a customs check point in Hong Kong. Authors compared three modes of data collection (see Table 5). First, all participants completed a FTF demographic survey and then were randomized to one of the three modes (in-person IVR, SAQ returned on-site in a self-sealing envelope, and SAQ to be completed off-site and returned via post) [18]. This article also included a comparison of two in-person self-administered questionnaires (in-person IVR compared with in-person SAQ; see Table 6). The authors found differential reporting of sensitive behaviors by mode. The low response rate (only 36% of men randomized to complete the postal SAQ returned the questionnaire) limits analysis of results. Item nonresponse rates and frequency of self-reported, sensitive sexual behaviors were statistically significantly different across the three methods for all reported questions. The IVR estimates were more similar to the remote (postal) SAQ than to the in-person SAQ. The postal mode reported socially desirable answers more frequently than the other two modes (both in-person and self-administered) [18]. This study’s authors suppose that subjective psychological responses, such as the perception of confidentiality, explain the lower report of undesirable behaviors in the self-administered in-person article survey compared with the other two modes.

**Table 4:** In-person interviewer administered compared with remote self-administered.

Author (year)	Country (sample type)^a^	Data collection #1 (sample size)	Data collection #2 (sample size)
Ballivian [14] (2013)	Honduras (dependent)	FTF^b^ (1500)	SMS^c^ (900)
Ballivian [14] (2013)	Honduras (dependent)	FTF (1500)	IVR^d^ (600)
Ballivian [14] (2013)	Peru (dependent)	FTF (1500)	SMS (677)
Ballivian [14] (2013)	Peru (dependent)	FTF (1500)	IVR (383)
Du [15] (2013)	China (dependent)	FTF (591)	SMS (591)
Li [19] (2013)	China (dependent)	FTF (177)	SMS (99)

^a^When participants were the same across modes, we classified the sample as dependent. Different participants across modes was labeled as an independent sample.

^b^FTF: face-to-face.

^c^SMS: short message service.

^d^IVR: interactive voice response.

**Table 5:** In-person self-administered compared with remote self-administered.

Author (year)	Country (sample type)^a^	Data collection #1 (n)	Data collection #2 (n)
Lau [18] (2000)	Hong Kong (dependent)	In-person IVR^b^ (1254)	Postal SAQ^c^ (556)
		In-person SAQ (606)	Postal SAQ (556)

^a^When participants were the same across modes, we classified the sample as dependent. Different participants across modes were labeled as an independent sample.

^b^IVR: interactive voice response.

^c^SAQ: self-administered questionnaire.

**Table 6:** In-person self-administered compared with a second mode of in-person self-administered.

Author (year)	Country (sample type)^a^	Data collection #1 (sample size)	Data collection #2 (sample size)
Lau [18] (2000)	Hong Kong (dependent)	In-person SAQ^b^ (606)	In-person IVR (1254)

^a^When participants were the same across modes, we classified the sample as dependent. Different participants across modes were labeled as an independent sample.

^b^SAQ: self-administered questionnaire.

#### Remote Self-Administered Compared With a Second Mode of Remote Self-Administered

Four articles make five comparisons of remote self-administered modes with a second mode of remote self-administered (see Table 7). Two articles compare Russian respondents’ answers on a Web survey on a mobile phone with a Web survey on a PC. The survey that compared a different population across the two modes did not find any difference in report of sensitive behavior indicators [26]. In the second study that was a cross-over experiment, 2 of the 5 sensitive questions, namely, alcohol consumption and income, were statistically significantly different [25]. The PC-based Web survey reported higher levels of alcohol consumption and higher income. The respondents reported higher trust in data confidentiality on the Web data collected on PC compared with the Web data collected on mobile phone. The authors also tested for interaction between gender and survey mode but did not find any statistically significant gender differences.

The World Bank study also compared SMS and IVR. The study found a higher attrition and lower survey-completion rate among IVR and SMS respondents compared with the other two modes [14]. Furthermore, panelists responding via self-administered mode were more likely to leave questions unanswered compared with CATI. Finally, SMS then IVR were estimated to be the least expensive options for data collection, compared with CATI and FTF.

In addition to the work in Peru, Honduras, and Russia, 1 article compared two modes of remote data collection. This survey was nested in a panel study in Nepal and compared CATI, and two types of SMS surveys. During single-sitting SMS interviews participants completed the survey at one time, and during module-design text interviews participants answered one question per day [24]. There were very few differences in results when comparing the two SMS modes. The modular survey did have higher nonresponse rate than the single sitting SMS survey, but the respondents in the modular design group found the survey to be significantly easier to complete than persons in the other two groups [24].

#### Remote Self-Administered Compared With Remote Interviewer-Administered

The research in Nepal compared the two aforementioned modes of SMS to CATI (see Table 8). They found that both text message modes increased the probability of disclosing sensitive information (eg, age of drinking onset, ever-smoking marijuana) compared with CATI, but mode did not impact the report of factual survey items (eg, marital status, age) [24]. The authors note that they are not sure whether sensitive behavior is reported more frequently via text due to decreased time pressure or increased privacy [24].

The final comparisons from the World Bank study is CATI compared with IVR and SMS. SMS, although the least expensive of the three modes, has twice the attrition rates as CATI [14]. Another important consideration about SMS from the World Bank study is that personal Internet access was reported more frequently via SMS. The World Bank study’s authors hypothesize that this could be caused by younger informants who are more likely to respond to an SMS survey compared with other ages. Reliability co-efficients for SMS range from .57 (Do you consider yourself poor?) to .87 (Do you currently have a television at home?) [14]. The Cronbach alpha for IVR is higher (at .86) than SMS, and item-level reliability has a smaller range from .79 (In the last 30 days have you access the Internet or not?) to .93 (Do you currently have a television at home?) [14].

**Table 7:** Remote self-administered compared with a second mode of remote self-administered.

Author (year)	Country (sample type)^a^	Data collection #1 (sample size)	Data collection #2 (sample size)
Mavletova [26] (2013)	Russia (independent)	Web MP^b^ (481)	Web PC^c^ (532)
Mavletova and Couper [25] (2013)	Russia (dependent)	Web MP (884)	Web PC (884)
Ballivian [14] (2013)	Peru (dependent)	IVR^d^ (383)	SMS^e^ (677)
Ballivian [14] (2013)	Honduras (dependent)	IVR (600)	SMS (900)
West [24] (2015)	Nepal (independent)	SMS: singular (150)	SMS: modular (150)

^a^When participants were the same across modes, we classified the sample as dependent. Different participants across modes was labeled as an independent sample.

^b^Web PC: Web on personal computer.

^c^Web MP: Web on mobile phone.

^d^IVR: interactive voice response.

^e^SMS: short message service.

**Table 8:** Remote self-administered compared with remote interviewer-administered.

Author (year)	Country (sample type)^a^	Data collection #1 (sample size)	Data collection #2 (sample size)
West [24] (2015)	Nepal (independent)	CATI^b^ (150)	SMS^c^modular (150)
West [24] (2015)	Nepal (independent)	CATI (150)	SMS singular (150)
Ballivian [14] (2013)	Peru (dependent)	CATI (384)	SMS (677)
Ballivian [14] (2013)	Honduras (dependent)	CATI (600)	SMS (900)
Ballivian [14] (2013)	Peru (dependent)	CATI (384)	IVR^d^ (383)
Ballivian [14] (2013)	Honduras (dependent)	CATI (600)	IVR (600)

^a^When participants were the same across modes, we classified the sample as dependent. Different participants across modes was labeled as an independent sample.

^b^CATI: computer-assisted telephone interview.

^c^SMS: short message service.

^d^IVR: interactive voice response.

## Discussion

### Principal Findings

This literature review synthesizes research that compares two or more modes of data collection in LMICs, with a special focus on remote data collection. We identified 10 articles that covered a range of modes and a variety of sampling methods. Three articles collected data in East Asia, 3 in Central and South America, 2 in Russia, 1 in South Asia, and 1 in Lebanon. No articles were identified that compared in-person self-administered and remote interviewer-administered surveys (eg, CATI). In-person self-administered includes a SAQ or a computer-assisted self-interview, with or without audio. Due to both heterogeneity of outcomes among the studies and the limited number of studies, this literature review is best positioned to present the current evidence rather than attempt to draw conclusions.

The most comprehensive study was conducted by the World Bank, where researchers made six comparisons across four modes of data collection (FTF vs IVR, FTF vs CATI, FTF vs SMS, IVR vs CATI, IVR vs SMS, and CATI vs SMS) in each of two countries [14]. This is the only study in the literature review to compare IVR versus CATI and IVR versus SMS. Comparing IVR and CATI is a particularly useful comparison because if the same sampling method is used for CATI and IVR, the impact of administration of interview can be better isolated and assessed. The finding that CATI and FTF estimates produced the best criterion validity when compared with the other modes in the study is consistent with findings from other studies included in this review.

Half of the studies enrolled participants FTF. Enrolling participants FTF mitigates one of the main benefit of remote data collection—reduced data collection cost. Only two of the studies used random digit dialing (RDD) and both were limited to landlines [16,17]. No articles in this review were conducted in SSA, but we expect an increasing amount of evidence will be emerging from the area.

A minority of articles explicitly compared the profile of respondents between the two modes of data collection. By comparing sample demographics to the target population, we will better understand the respondent bias a mode may introduce. Ferreira et al found that groups with higher telephone ownership or coverage were more likely to report better health [16] and the World Bank study identified young people as more likely to respond to a SMS survey [14]. Other key information, including cost, length of the questionnaire, and the reliability of measures were not reported consistently but would provide important implementation information. It is imperative to use American Association for Public Opinion Research Reporting Guidelines so that survey metrics are comparable [27].

The impact of mode on reporting sensitive behaviors in LMIC is discrepant [12]. A meta-analysis that compared 15 data sets (which included no forms of remote data collection), mostly comparing FTF and audio computer-assisted self-interview found that non-FTF methods did not consistently produce a significant increase in the reporting of 4 sensitive questions [12]. In this literature review, only 3 articles offered a straight-forward comparison of nondesirable behaviors. In 2 of the articles, remote data collection elicited higher report of nondesirable behaviors compared with in-person data collection [18,20]. The article that compared CATI, single-sitting and modular-design SMS found that the SMS respondents reported more socially undesirable behaviors compared to CATI [24].

Considering that all but one article in this literature review had been published in the past 5 years, we anticipate an increase in publications comparing modes of data collection in the coming years. As evidence continues to emerge, research designed to isolate the cause of differences in measures between modes should be a priority. For example, researchers should ask direct questions around the impact of increased privacy, greater anonymity, or greater convenience of remote data collection. Only 3 of the articles in this literature review included such questions [24,25,26]. By eliciting participant’s opinions about the different modes, discrepancies can be better explained. Country context, such as literacy levels, mobile phone ownership, and network coverage are particularly important to note when considering remote data collection in LMICs. Therefore researchers should include aforementioned information in publications so that conditions can be considered. Furthermore, reporting factual, sensitive, or perceptual questions, as well listing the type of question (such as multiple choice, numeric, text) all provides pertinent information for decision making.

Although studying mode effect is an important aspect of remote data collection research, sampling is equally pertinent. RDD functions without a sampling frame in many countries, which means after identifying all mobile network operator prefixes in a country, numbers are randomly generated [28]. It is unknown whether RDD can consistently produce nationally representative estimates of a health outcome or which mode is best suited for RDD. Six of the 10 studies in this literature reviewed enrolled participants in-person. The advantage of FTF enrollment is that the research team has a reference standard against which the remote data collection tool is measured. When enrolling participants for FTF, asking how many mobile phone numbers each participant has and their estimated network coverage helps to estimate how representative the sample will be. Finding out the preferred language of survey while enrolling a participant will allow the first follow-up contact to be in the respondent’s language of choice, negating the need for a language question and likely increasing the response rate. It is unknown whether RDD is more likely to enroll respondents who are hard to reach in FTF surveys, but this hypothesized advantage should be assessed. RDD and remote data collection generally have the advantage of faster collection of data than a FTF survey, thus making this approach particularly useful during a crisis.

To isolate a superior method of data collection, studies that compare more than two modes of remote data collection are preferred. Only three studies in this review compared more than two modes [14,18,24]. Specifically, future research should follow mHealth guidelines [29], incorporating a factorial design where possible. As a minimum, it would be advantageous for authors to identify which questions are sensitive in their context so that mode effects for sensitive questions can be compared, even if the subject matter is different.

### Limitations

We note three main limitations to this literature review. First, the small number of studies (n=10) that compared two modes of data collection or more (n=25 comparisons), made it difficult to draw conclusions. The included research used a wide variety of data collection modes and sampling techniques and covered a plethora of topics and populations, thus negating the ability to make conclusions. Furthermore, the nonlinear relationship of effects can make pattern identification a challenge [11]. Second, owing to the inherent limitations of searching for grey literature, our search strategy could have missed important articles. A third limitation pertains to the inconsistent reporting of key survey metrics as well as lack of a formal statistical test to analyze the variation between the results of each article. Regardless of these limitations, this literature review contributes to efforts to characterize current evidence on the effect of remote data collection mode on data quality in LMICs.

### Conclusions

Due to the nascent state of remote data collection in LMICs, several research areas merit further investigation. The advantages of remote data collection are presented, but a superior mode for a population has yet to be established due to a dearth of evidence. We encourage randomized control trials with multiple arms to identify a mode appropriate for the context. Ultimately, researchers must balance the desire for more efficient, cost-effective data collection methods with study aims and the limitations of a novel mode of data collection.

## Abbreviations

CATI: Computer-assisted telephone interview
FTF: face-to-face
HIV: Human Immunodeficiency Virus
IVR: interactive voice response
LMIC: low- and middle-income countries
PC: personal computer
RDD: random digit dialing
SAQ: self-administered questionnaire
SMS: short message service
SSA: Sub-Saharan Africa
